# Transactions of the Amer. Med. Association of Southern Central N. Y.

**Published:** 1852-03

**Authors:** 


### ARTICLE V.

*Transactions of the Medical Association of Southern Central New York, at the Annual Meeting held at Bringhampton, June*, 1847.

This is a volume of 121 pages, octavo, made up principally of Reports and original papers mostly relating to the condition and practice of medicine in the district included by the limits of the Association, and cases that have occurred in the practice of different members.

It shows a commendable zeal on the part of the members and speaks well for the ability with which they are pursuing the study and practice of medicine.

